# A Few Hints to Students

**Published:** 1893-02

**Authors:** William Rushton


ARTICLE VII.

### A FEW HINTS TO STUDENTS.

BY WILLIAM RUSHTON, L. D. S., ENG.

**Read at the Students' Society, National Dental Hospital, London.**

*Mr. President and Gentlemen:*—It was with great pleasure that I acceded to the request of your secretary to read a paper to-night, because I thought that an evening might be profitably spent in thinking over and discussing a few points that crop up in every-day practice.

To many here these remarks may seem of a very elementary nature, but I hope they may bear them with patience, remembering that if it is good to "seek fresh woods and pastures new," so it is also sometimes good to "repeat a thrice-told tale." We are all students, and I hope we will remain so to our dying day. It is only fashionable young ladies who "finish their education."

Firstly, I cannot too strongly impress upon you the importance of cultivating a systematic method. Acquire the habit of going through a fixed routine upon every mouth which presents itself. The first thing to do is to ask the patient the reason of his requiring your aid, and then, by a series of intelligent questions, gain a rough idea of what is the matter.

After this has been done, proceed to examine the mouth in a systematic manner. A good rule is always to begin at the left upper molars, look out for caries on the crown, mesial, distal, and buccal surfaces; if you see an opaque white patch where two teeth meet, break down with a chisel without hesitation, though you may see no caries. Examine the bicuspids in the same way. Look out for caries in the canine on the mesial and distal surfaces near the gum, and erosion on the labial surface. In the incisors, in addition, examine the pit on the lingual aspect where the two ridges of the cingulum join, and so work round to the right upper wisdom. Also note which teeth are filled, whether any look like dead teeth, any signs of abscesses, pyorrhœa alveolaris, looseness or tartar. Then turning your mirror on to the right lower wisdom, come right round to the side you started from.

Now you have done two important things: you have learnt your patient's sensations, and you have made yourself acquainted with the tooth or teeth that may or may not cause those sensations; and it is in diagnosing differentiating and scientifically relieving that your skill comes in.

Three instruments should always be at hand in examining the mouth: the mirror, the probe, and the dressing forceps, with cotton wool, or, still better, bibulous paper.

The mirror should be held in the left hand in such a manner that it reflects light on to the tooth under examination, and I may here remark that the mirror which has been scratched by corundum or disc should be kept only for such times as you are using corundum, disc, or pumice, and that for filling or examination purposes you should keep another and a faultless glass. Another point in the mirror is, that it should have a stiff handle. Those folding mirrors which I have occasionally seen are useless and vexatious; it is impossible to press away the cheek or control the tongue with them. I am told they are handy for the pocket; then by all means keep them in the pocket.

The dressing forceps grasping the cotton wool should be held in the right hand, and should accompany the mirror in its travels, clearing away saliva and mucus. Then lay down the dressing forceps, and go round each tooth with your probe, looking out for caries in the manner described. The dressing forceps will find out any caries that has made considerable progress, while the probe will find out the beginning of caries and hidden cavities between teeth. The probe is an instrument which should be used with great circumspection. If you plunge it into an exposed pulp, or into the gum, your patient is apt to lose confidence in your skill, and gentle treatment, and the possession of the patient's confidence is half the battle in dentistry.

Having asked your patient a few leading questions, and having examined his mouth, you can as a rule make a pretty shrewd guess as to what is the cause of his trouble.

Let us take a typical example of two.

A patient comes to us complaining of toothache. Toothache may be caused by any of the following :

Sensitive dentine, (*a*) caries.

| | | |
|---|---|---|
| Sensitive dentine, | (*b*) | through erosion. |
| " | (*c*) | metal filling. |
| " | (*d*) | recession of gum. |
| " | (*e*) | attrition. |
| " | (*f*) | mechanical injury. |
| Irritation of pulp, | (*a*) | through exposure. |
| " | (*b*) | through the action of a filling. |
| Irritation of pulp, | (*c*) | intrinsic calcification. |
| " | (*d*) | mechanical injury. |
| Inflammation of pulp, | (*a*) | through exposure. |
| acute and chronic, | (*b*) | tnrough a filling. |
| with or without periostitis. | (*c*) | through mechanical injury. |

Irritation or inflammation of the gum through the pressure of food or a filling.

Periostitis, through a putrid pulp or mechanical irritant, such as the bristle of the tooth-brush, etc.

Alveolar abscess.

Exostosis.

Painful eruption of wisdom-teeth, irritation or disease of the central nervous system, and, I daresay, some more which I have omitted.

So here we have a variety of causes for the disease on account of which our services are sought; it is our business to find out which of these is the enemy in each particular case, and to combat it.

Now, never do what I have sometimes seen students do—viz., find out a carious tooth and apply the rubber dam without asking a single question (and by applying the rubber dam, doing away with their patient's ability to answer questions) not knowing even whether the tooth is alive or dead. Before you apply the rubber, or start excavating, try and get to know all about that tooth.

Q. Where have you felt the pain ?

A. The patient usually places a finger near the seat of pain; this may be a guide, but is not a criterion, as their feelings often misguide them.

Q. How long has the tooth hurt you?

A. For a fortnight, on and off: but it has been getting worse.

Q. Does it keep you awake at night?

A. I did not sleep a wink last night.

Q. Any swelling or gum-boil?

A. No; but very painful to touch.

Now, by these questions, we judge that there must be acute inflammation, though whether caused by the pulp only, or the periosteum only, or both, we are not quite sure. True, we could see and feel for ourselves if the tooth is very painful, or if there is any swelling, but if we can gain the same information without giving our patient pain, it is our duty to do so.

We examine the mouth as already mentioned, and find an upper bicuspid, the anterior wall of which has broken away, the cavity filled with food and *debris*, a bright red margin around the neck and the tooth slightly loose.

This is one of the most painful conditions with which we have to do, and we cannot be too gentle and considerate to our patient.

Syringe the cavity out with tepid water, dry it gently with bibulous paper, and proceed with sharp spoon excavators to take away the spongy carious dentine. See that your instruments are sharp, cut always from the direction of the pulp, never towards it, and don't fiddle about; let each stroke be decided. Commence at the periphery and clear away all the dentine, save that which covers the pulp chamber. In that pulp chamber you may expect to find one of two things; either an inflamed pulp, or a disorganized one. You are not *quite* certain which, as the pain of your excavation may have been due to the periostitis present, not only to nerve pain. Take a pellet of cotton wool, dipped in cold or hot water, and place in the cavity. No pain means a dead pulp, a start of pain means a live pulp. Slight pressure with a burnisher, with a start of pain, means a live pulp, as the pressure is felt by the pulp through the spongy dentine. However, whether alive or dead, your duty is the same—to expose the pulp chamber. This must be done fearlessly, with sharp instruments and a bold hand; and while exposing, do so thoroughly, for the smaller the exposure, the more painful is the process of devitalizing, and *vice versa.* After two or three sharp strokes, you see a bright red spot from which issues more or less blood, and you know the patient has been suffering from an acutely inflamed pulp with periostitis. Your duty is of course to apply the rubber dam; if possible, though not if too painful, devitalize, extract the pulp later on, and fill in due course.

Let us take one more case. A patient asks you to remove an upper molar, you examine the mouth and come to the conclusion that the upper molar, though somewhat carious, is not the cause of the pain.

Q. Where do you get the pain?

A. It runs from that tooth right up the side of my face.

Q. Does it pain you at any particular time?

A. Yes; when I drink hot tea, and when I lie down at night.

Q. Does cold hurt you?

A. No; sometimes I hold cold water in my mouth and it eases the pain.

Q. You have a lower tooth stopped; do you have any pain from that?

A. I may have, but the whole side of my face seems so painful that I don't know where it comes from.

Q. How long ago was that tooth stopped?

A. Three months.

Q. Was it painful?

A. No, not very.

We tap the upper tooth with the handle of the mirror—no pain; we do the same with the lower, and the patient shrinks a little, but says the pain is nothing to speak of.

We get a large pellet of cotton wool, dip it in cold water, and place it on the filling, which is amalgam—no pain. We then get a large ball ended burnisher and place it in the flame till it approaches dull red heat. Then dry the tooth and place the hot burnisher on the filling. The patient does not feel it immediately, but in a few seconds says: "You have made it ache," or some such remark. Then you feel you are on the track, and at once proceed to drill out the amalgam. When this has been removed, you may find some pus oozing into the cavity. Perhaps you will think that as pus is present, you have a dead tooth to deal with and proceed to pass up a bristle or barb. If you do, you will wish you had not, for you will find that the lower end of the pulp only is dead, while the apical portion is alive. Thus we find that chronic irritation (perhaps present before the filling was inserted or produced by the filling) has been going on, getting worse, and suppuration has commenced. If the inflammation had been more acute, the pain would have been localized by the patient to the correct tooth, and there would have been most probably no wandering neuralgic pain, but very likely more periostitis.

We thus have taken two cases: in the first, we find how our patient may help us in our diagnosis; in the second, we see how they may at first misled us.

Always work with sharp tools. How can you tell they are sharp? Turn the edge towards the light; if you can see a line of light reflected from the edge, it wants sharpening till that line can no longer be seen. Have a stone by you and get into the habit of looking if your instrument be sharp, and if not, of sharpening it.

If you have the misfortune to wound a pulp which has never previously ached, dust immediately the wound with iodoform, varnish with copal, mastic, or carbolized resin, wait until the varnish is dry, and then flow on thin oxyphosphate. Never use gutta-percha in these cases, as the heat and pressure may be injurious.

If a pulp is sufficiently inflamed to give its owner a wakeful night, or even a prolonged pang of toothache, my rule is to devitalize.

White's nerve fibre, loosely twisted around a broach, is very convenient for devitalizing apices of pulps. It is always ready, easily introduced, easily withdrawn, and is efficacious.

I once put in a very satisfactory gold filling, and was surprised and annoyed to see my patient return in a few days with acute periostitis. I found it was caused by my carelessly leaving the ligature round the tooth after removing the rubber dam.

On another occasion, a similar incident occurred, and found that this time the periostitis was caused by my leaving too much gold in the filling, which was therefore gagging the mouth, and the tooth receiving consequently tremendous pressure. *Verb. sap. sat.*

Mix (in ordinary cases) your oxyphosphate stiff. Thin fillings soon wash away. Have your tooth perfectly dry and ready. Mix quickly, always adding your powder to your liquid. If you find you have added too much powder, or have not enough liquid, never add more liquid, but start afresh. By adding the liquid to the half-set cement you alter the character of the preparation. Oxyphosphate mixed thickly and quickly and introduced at once has enough adhesive power for all ordinary purposes, it sets in a few seconds and forms a much more durable filling than those introduced in a "creamy" state. Do not touch your cement filling until it is set; then trim off, always working towards the edge of your cavity, never from it.

The golden rule for gold fillings is, "Take care of the edges (especially the cervical), and the middle will take care of itself."

I once asked a student whether he thought a certain cavity was of a proper shape to hold gold. His reply was, "It will hold amalgam." Now if a cavity is of a shape to hold amalgam, it will also be of a shape to hold gold. There seems to be an idea among students that amalgam (being a plastic filling) adheres to the wall of the cavity after the manner of an oxyphosphate. Of course such is not the case, both amalgam and gold fillings are retained by the shape of the cavity. The details may be modified in each instance, but the fundamental rule is the same. If the same pains were taken over the preparation of cavities and their filling with amalgam as there is with gold, we should see less of failures in that respect.

No matter with what substance you are filling a tooth, pack it in bit by bit. I know of no exceptions to this rule, except perhaps pin-holes filled with gutta-percha. Gold, whether used cohesively or non-cohesively, must of necessity be packed in piece by piece, and (in reason) the smaller the pieces the better; the less likely they are to rock and the better they are to condense and cohere.

Amalgam should also be packed bit by bit. The best carrier I know is one of this shape, having a right-angled serrated end. Hold your amalgam between the tips of your thumb and index finger tightly, then press your carrier through the outside portion of the amalgam, and it then bears away a pyramidal mass which clings tightly to it. This can be turned upside down and carried to any part of the mouth; with mine I can fill a distal cavity in an upper wisdom tooth with very little more difficulty than a mesial cavity in a first lower molar.

Having taken a small mass of amalgam, place it in your cavity and burnish well with a small instrument into your undercut or floor, and so on, until the cavity is full, using a somewhat larger burnisher as you proceed. Finish off by absorbing excess of mercury with tin-foil or adding excess of dry amalgam and burnishing off.

In every operation upon the mouth (except extraction with forceps) use your instrument resting on your finger or thumb from a fixed point, the most useful fulcrum being a neighboring tooth or the tooth you are working upon. By this means your hand is steady, you get increased power and you have perfect command over your instrument, which, if it slips, cannot slip far enough to do any damage. Are you chiseling an upper molar? Rest your index finger or thumb (whichever you are working from, the front or the back) upon the tooth, and grasping the chisel firmly with the other fingers, let it work upon the guiding finger or thumb, and while these latter are steady, your instrument is safe. The same rule applies to engine burs, excavators, and scalers, and in extraction with the elevator.

I have found that new students in purchasing instruments generally err in choosing them of a large size. These are useful in some cases, but always remember that, while you can get a small excavator or bur into a large cavity, you cannot get a large one into a small cavity; and in conservative dentistry small cavities are, or should be, the rule.

Never apply the rubber dam until you have roughly excavated and shaped the cavity, as you run the chance of wounding the rubber with your instrument, and so are obliged to re-apply it. By trimming your enamel edges, you obviate the risk of cutting your rubber and your ligature passing them up.

Gentlemen, this has been a rambling paper of odds and ends. I trust it has been of some service to some of you, but if it has no other effect than that of eliciting remarks and discussion, it will not have been jotted down in vain.—*British Journal of Dental Science.*

